# Supplementary material for: Multiple Viral Infections Detected in Phytophthora condilina by Total and Small RNA Sequencing
Source: Viruses. 2021 Apr 4;13(4):620. doi: 10.3390/v13040620 (PMC8067226; doi:10.3390/v13040620)
Supplement: Supplementary file 1 [file viruses-13-00620-s001.pdf]

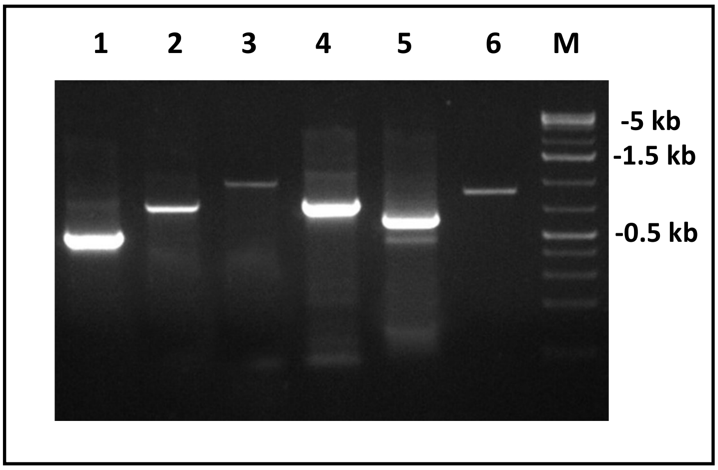

Figure S1. Amplification of the described bunyaviruses using dsRNA as template in the cDNA synthesis. 1-PcoNSRV2 (amplicon 511 bp), 2-PcoNSRV3 (amplicon 740 bp), 3-PcoNSRV1 (amplicon 983 bp), 4-PcoNSRV4 (amplicon 743 bp), 5-PcoNSRV11 (amplicon 623 bp), 6-PcoRV1 (amplicon 861 bp).

Figure S2. Graphical representation of the inserts in the small RNA library showing two main peaks at 21 and 25 nts.

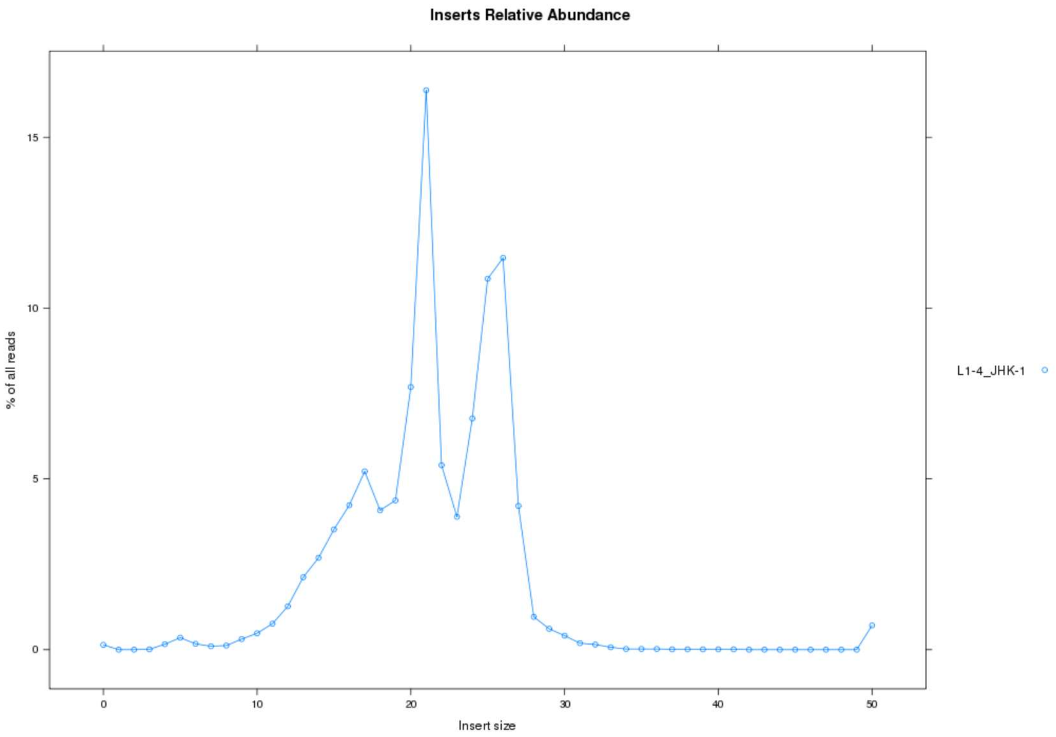

SUPPLEMENTARY TABLES

Table S1. Primers used for viral presence confirmation and RACE

Name[N]	Virus	Sequence (5'- 3')	Position in genome (5′-3′)	Amplicon size (bp)
DN95_R	PcoRV1	GAGAACCAATACTCAGGAACCGT	2254-3114	861
DN95_F		ACAGTGGGCGTATTTCTGTAAGT		
DN95_RACE_R_5'	PcoRV1	TCCGTCAGTAGCGCTACTCTGCCG	4862	989
DN95_RACE_F_3'		CGAGCATGGGAGGTGGTGGGATCT	894	894
DN789_F	PcoRV2	CAACCATTCTTGCACTTCAGGAG	3179-4108	930
DN789_R		TGAGATCGAGTTGCTTTCCAAGA		
DN789_RACE_R_5'	PcoRV2	GCCAGGTACGTCCGACCGACTCTT	489	489
DN789_RACE_F_3'		CCCCCGAGCCCAACCCAGAACTAG	5628	613
DN6573_R	PcoNSRV1	AAAGCATTTGTGCGTATGGAACA	2310-3273	983
DN6573_F		ACCGAACACTCTTTCTGCAATTG		
DN278_ R	PcoNSRV2	TTCGGCCGTAAGTCTCTTGTTTA	4023-4533	511
DN278_F		TGGGAATACCGAACACTCTTGTT		
DN157_R	PcoNSRV3	GCATATTGTACCTAACGTGCACC	1517-2256	740
DN157_F		TACACATACCGGGAAAGCTGAAA		
DN32_g2_R	PcoNSRV4	CCAATGAGCTGGAATTCCGATTC	1947-2689	743
DN32_g2_F		GGGCAGCACAGACATCAAATATC		
DN5725_R	PcoNSRV5	TCGAGCTCTTTTCAAATGGAGGA	3290-3835	546
DN5725_F		GAAGGTCACACTTTTTAGTGCCC		
DN3746_R	PcoNSRV6	TTCTCTCTTCAAGTGCCACTCTC	4527-5124	598
DN3746_F		GGACAGAGCAACGTTTCATATCG		
DN7515_R	PcoNSRV7	AGCAAGTTCGGGTTCATTTTCAG	3258-4219	961
DN7515_F		CAACAGATGTTACCTTTGCAGCA		
DN143_R	PcoNSRV8	GCTCTTGTGAAAGACCTCTCAGA	1859-2506	648
DN143_F		GTTTGCGCTGTATCATACTCCAC		
DN43_g2_R	PcoNSRV9	CAAATCGCATTTATTCTCCCGCT	779-1651	873
DN43_g2_F		GACAGTAGACCAGGTAATAGGCG		
DN3737_R	PcoNSRV10	TCGACACACCTGCAAGATTTTTC	1849-2804	961
DN3737_F		AATGCTAAAGCTCTGGTCAGTGA		

DN34_g1_i1_R<br>DN34_g1_i1_ F	PcoNSRV11	CTACCCAAGACATATACACGCGA<br>CTGTTGAGCCTTAATCATCGCTG	6751-7373	623
DN47_RE<br>DN47_FW	PcoNSRV12	GGTTTGGGGCTTAATGATGACAAA<br>CTGTGTACCAACCATTACCTGAGA	3903-4540	638
DN32_g1_R<br>DN32_g1_F	PcoNSRV13	ACACCAAAATTGACGAGCTTCTG<br>TTGCTGCATCTTTGACACTTGAG	4382-5209	828

[N]Corresponding contig sequence obtained in TRINITY.

Table S2. Size distribution of small RNA inserts from *Phytophthora condilina* isolate BD661

Insert range	0	1-17	18-26	27-50
reads	67,327	10,344,387	34,109,189	3,575,124
% reads	0.14	21.51	70.92	7.43

Table S3. Pairwise identities of the bunyavirus sequences at nucleotide level. Distances are based on the MUSCLE alignment of all the 13 sequences, which had 11,040 sites.

	PcoNS RV3	PcoNSR V12	PcoNSR V10	PcoNS RV8	PcoNS RV5	PcoNS RV1	PcoNS RV2	PcoNS RV9	PcoNS RV6	PcoNSR V13	PcoNS RV4	PcoNSR V11
**PcoNSR V12**	20.31											
**PcoNSR V10**	20.40	38.02										
**PcoNSR V8**	20.83	38.03	44.57									
**PcoNSR V5**	20.35	38.05	44.66	51.46								
**PcoNSR V1**	17.87	21.23	24.12	22.90	22.91							
**PcoNSR V2**	20.39	25.48	27.45	28.00	27.71	27.43						
**PcoNSR V9**	20.78	26.49	29.24	29.30	29.21	27.52	32.14					
**PcoNSR V6**	21.08	26.46	29.14	29.05	28.64	27.02	31.31	53.21				
**PcoNSR V13**	21.02	27.36	29.45	29.53	28.95	26.81	31.92	38.46	37.68			
**PcoNSR V4**	20.25	27.17	29.25	28.94	28.81	27.56	32.41	39.29	38.09	44.95		
**PcoNSR V11**	21.13	27.81	29.76	29.25	29.42	27.24	32.12	39.27	39.19	42.92	45.27	
**PcoNSR V7**	21.85	28.02	29.75	29.72	29.87	27.30	33.46	39.07	38.81	42.90	44.24	49.10

Table S4. Pairwise identities of the bunyavirus sequences at protein level. Distances are based on the MUSCLE alignment of all the 13 sequences, which had 3,689 sites

	PcoNSR V2	PcoNSR V3	PcoNSR V1	PcoNSR V9	PcoNSR V6	PcoNSR V13	PcoNSR V4	PcoNSR V11	PcoNSR V7	PcoNSR V12	PcoNSR V10	PcoSNR V8
**PcoNSR V3**	6.20											
**PcoNSR V1**	8.27	6.48										
**PcoNSR V9**	10.51	8.80	10.82									
**PcoNSR V6**	10.33	8.50	10.19	45.12								
**PcoNSR V13**	10.23	8.49	10.65	24.91	24.44							
**PcoNSR V4**	10.84	8.67	10.87	26.11	24.12	32.63						
**PcoNSR V11**	10.17	8.52	10.80	25.45	25.38	29.94	32.81					
**PcoNSR V7**	10.50	9.26	10.28	25.53	25.11	29.87	32.78	36.62				
**PcoNSR V12**	9.16	7.59	11.32	13.61	14.13	12.87	12.79	12.80	13.32			
**PcoNSR V10**	9.78	8.46	11.73	14.73	14.29	14.08	13.13	14.12	14.41	26.68		
**PcoSNR V8**	10.64	8.69	10.87	15.07	15.03	14.85	14.74	15.71	15.23	26.55	30.28	
**PcoNSR V5**	10.64	8.23	10.59	15.58	14.63	14.18	14.21	15.52	14.54	26.44	30.77	41.83
